# Epithelial cell–based multi-omics integration identifies SPINK5 and SRI as novel diagnostic biomarkers for ulcerative colitis

**DOI:** 10.3389/fphar.2026.1797539

**Published:** 2026-06-10

**Authors:** Xingwang Li, Zhanzhen Liu, Zheng Ge, Yuewei Li, Kunhou Yao, Xuhong Lin, Xiaoming Liu, Hongping Xia

**Affiliations:** 1 Department of General Surgery, The First Affiliated Hospital of Xi’an Jiaotong University, Xi’an, China; 2 Department of General Surgery (Colorectal Surgery), Huaihe Hospital Affiliated to Henan University, Kaifeng, China; 3 Department of General Surgery (Colorectal Surgery), The Sixth Affiliated Hospital, Sun Yat-sen University, Guangzhou, China; 4 Guangdong Provincial Key Laboratory of Colorectal and Pelvic Floor Diseases, The Sixth Affiliated Hospital, Sun Yat-sen University, Guangzhou, China; 5 Biomedical Innovation Center, The Sixth Affiliated Hospital, Sun Yat-sen University, Guangzhou, China; 6 Department of Gastroenterology, Huaihe Hospital Affiliated to Henan University, Kaifeng, China; 7 Zhongda Hospital, School of Medicine, Advanced Institute for Life and Health, Southeast University, Jiangsu, China

**Keywords:** diagnostic biomarkers, machine learning, serine protease inhibitor kazal type 5, sorcin, ulcerative colitis

## Abstract

**Objectives:**

Ulcerative colitis (UC) is a chronic inflammatory bowel disease characterized by epithelial barrier dysfunction, yet reliable epithelial-derived diagnostic biomarkers remain elusive.

**Methods:**

We employed an integrative multi-omics approach, combining single-cell RNA sequencing (scRNA-seq) and bulk transcriptomics from public datasets. Epithelial cell-specific signatures were identified and intersected with differentially expressed genes to pinpoint candidate markers. Four machine learning algorithms—LASSO, Random Forest, Support Vector Machine and K-Nearest Neighbor (KNN)—were applied for robust feature selection. The diagnostic model was validated in external cohorts, and its association with the immune microenvironment was assessed using CIBERSORT. Finally, key findings were experimentally confirmed in clinical UC tissues via qPCR, Western blot, and immunohistochemistry.

**Results:**

scRNA-seq revealed significant transcriptional remodeling of epithelial cells in UC. Cross-algorithm analysis consistently identified SPINK5 and SRI as the most robust diagnostic biomarkers. A model built on these two genes demonstrated exceptional performance, with an area under the curve (AUC) > 0.90 in both training and external validation sets. Immune infiltration analysis revealed a pro-inflammatory shift in UC and delineated distinct correlations of SPINK5 and SRI with specific immune cell subsets. Experimental validation confirmed significant upregulation of SPINK5 and downregulation of SRI in UC patient tissues at both the mRNA and protein levels.

**Conclusion:**

Our study underscores the pivotal role of epithelial dysfunction in UC pathogenesis. By integrating multi-omics and machine learning, we have established SPINK5 and SRI as promising candidate diagnostic biomarkers, providing candidate biomarkers with potential utility for UC diagnosis and a basis for future studies on disease stratification.

## 1 Introduction

Ulcerative colitis (UC), a chronic relapsing inflammatory bowel disease (IBD), involves a multifactorial pathogenesis including genetic susceptibility, disruption of the intestinal barrier, immune dysregulation, and metabolic abnormalities. Clinically, the lack of sensitive and specific molecular diagnostic markers poses challenges for early diagnosis, disease activity monitoring, and individualized therapy (Zheng, 2023; Serigado et al., 2022). In recent years, advances in single-cell RNA sequencing (scRNA-seq) have significantly enhanced our understanding of IBD tissue heterogeneity, cellular composition, and transcriptional states, providing a powerful tool to dissect the cellular-level pathogenesis of UC (Liu et al., 2024; Song et al., 2025).

Although previous studies have employed scRNA-seq to map the immune cell landscape in IBD, identifying specific inflammatory monocytes and T cell subsets, the molecular specificity and potential diagnostic significance of epithelial cells—key players in mucosal barrier damage and repair—remain underexplored (Wang et al., 2025). Epithelial cells not only maintain physical barrier integrity but also modulate immune cell infiltration via cytokine and chemokine secretion. Dysfunction of these cells is considered a central contributor to UC relapse and chronic inflammation (Wu et al., 2024). Meanwhile, machine learning (ML) techniques have been increasingly applied to clinical and transcriptomic data analysis. Recent studies have used algorithms such as random forest, LASSO regression, and support vector machines to construct diagnostic or disease activity prediction models for UC, thereby identifying potential key genes and cell types (Chen et al., 2025; Chen G. et al., 2024; Wu et al., 2025). For example, some studies combined weighted gene co-expression network analysis (WGCNA) with machine learning to identify candidate UC biomarkers, which were further validated in independent cohorts (Chen Y. et al., 2024). Others integrated scRNA-seq with machine learning to reveal cell-specific heterogeneity in tryptophan metabolism in UC, identifying key genes such as CTSS, S100A11, and TUBB (Wu et al., 2024; Chen G. et al., 2024).

In addition, multi-omics approaches, such as transcriptomics combined with proteomics, have been applied to investigate UC-specific immune microenvironments. For instance, in UC patients with cytomegalovirus (CMV) infection, combined transcriptomic and proteomic analyses uncovered crucial immune-related markers, providing new insights for diagnosing complex clinical subtypes (Bao et al., 2023). Furthermore, in fibrosis-related studies, scRNA-seq and machine learning analyses have identified key genes associated with fibrotic cells (e.g., fibroblasts) such as BIRC3 and ANXA1, and constructed UC diagnostic models, offering potential targets for predicting anti–TNF-α treatment response.

Epithelial-immune interactions are central to UC pathogenesis. Single-cell studies have identified TREM1^+^ inflammatory monocytes as significantly enriched in IBD, suggesting that they may amplify inflammatory responses via specific signaling pathways and serve as potential therapeutic targets (Tang et al., 2023). Moreover, spatial transcriptomics combined with machine learning has been employed to construct interpretable predictive tools, enabling subtype classification or disease progression prediction in IBD by resolving intestinal cellular spatial organization and microenvironment features (Saini and Acharjee, 2025).

Despite these advances, most studies have focused on immune cells or metabolic pathways, with few systematically exploring the diagnostic potential from the epithelial cell perspective. Considering the dual role of epithelial cells in maintaining mucosal integrity and mediating inflammatory processes, identifying epithelial cell–related genes at single-cell resolution and constructing diagnostic models using machine learning represents an important avenue to address this gap. While immune and stromal cells are equally important in UC pathogenesis, this study focuses on epithelial cells as a complementary perspective to existing immune-centric biomarker studies.

SPINK5 (serine protease inhibitor Kazal-type 5) is an important regulator of epithelial barrier integrity. It functions by inhibiting serine proteases that are involved in epithelial desquamation and inflammatory signaling. Dysregulated protease activity has been implicated in mucosal barrier disruption and excessive immune activation in inflammatory diseases. Therefore, altered expression of SPINK5 may directly influence epithelial stability and contribute to the pathogenesis of ulcerative colitis. SRI (sorcin) is a calcium-binding protein that plays a critical role in intracellular calcium homeostasis, cellular stress responses, and apoptosis regulation. Calcium signaling is essential for epithelial cell survival, repair, and barrier maintenance. Dysregulation of SRI may impair epithelial resilience under inflammatory stress conditions, thereby contributing to epithelial dysfunction and delayed mucosal healing in UC.

In this study, we first identified UC epithelial cell–specific genes using scRNA-seq data and extracted candidate genes by intersecting differentially expressed genes with bulk RNA-seq datasets. Subsequently, LASSO, random forest, support vector machine, and K-nearest neighbor (KNN) algorithms were applied for cross-selection of candidate genes to identify potential diagnostic markers. We further validated model performance in independent external cohorts and explored the association between key genes and the mucosal immune microenvironment through immune infiltration analysis. Finally, the expression of these markers was validated in human tissues using Western blot, immunohistochemistry, and qPCR. Our goal is to explore epithelial-derived candidate biomarkers for UC diagnosis.

## 2 Methods

### 2.1 Data acquisition

UC-related datasets were retrieved from the GEO database, including GSE92415 and GSE87473. From GSE92415, which contains samples collected before and 6 weeks after golimumab or placebo treatment. To reduce the potential influence of treatment on gene expression profiles, only 21 normal samples and 87 baseline UC samples collected before golimumab or placebo treatment were included as the training cohort. GSE87473, comprising 21 control samples and 106 UC samples, was used as an independent external validation cohort and was not involved in feature selection or model construction. Despite baseline filtering, residual heterogeneity in disease severity, duration, and prior medication may persist due to incomplete clinical annotation in public datasets. Additionally, the single-cell RNA-seq dataset GSE231993, including four UC samples, was obtained for epithelial cell analysis. The two bulk datasets were processed independently without batch correction or joint normalization to avoid cross-cohort data leakage.

### 2.2 Single-cell analysis

Single-cell RNA-seq data were processed using Seurat (v5.0). Quality control excluded cells with <200 detected genes or >20% mitochondrial gene content, and genes expressed in fewer than five cells were removed. Gene expression data were normalized using the LogNormalize method with a scale. factor of 10,000. The top 2,500 highly variable genes were selected using the variance-stabilizing transformation method to retain the major biological variation while reducing the influence of low-variance genes and technical noise. Standardization and principal component analysis (PCA) were then conducted.

Clustering was performed based on the first 15 principal components using a nearest-neighbor graph, with the clustering resolution set to 0.6. This resolution was selected to identify the major cell populations while avoiding excessive subdivision of closely related cell states. UMAP was used for dimensionality reduction and visualization. Differentially expressed cluster markers were identified with FindAllMarkers (min.pct = 0.2, |log_2_FC| > 1, FDR <0.05, Benjamini–Hochberg correction). Cell types were annotated using SingleR with reference to the Human Primary Cell Atlas (celldex package). Cell type proportions across samples were quantified and visualized using bar and alluvial plots to assess compositional differences. The single-cell dataset was used to identify epithelial cell-associated genes and provide cell-type-level information for subsequent bulk transcriptomic analysis.

### 2.3 Differential expression and functional enrichment of intersection genes

Differential expression between UC and control groups was analyzed using the limma R package, employing a linear model with empirical Bayes moderation for robust variance estimation. Genes with |log2FC| > 1 and FDR <0.05 were considered significantly differentially expressed. Heatmaps and volcano plots were generated using pheatmap and ggplot2 to illustrate expression patterns and statistical significance. DEGs were then intersected with epithelial cell–specific genes identified from scRNA-seq, and the resulting gene set was used for downstream functional enrichment and biological interpretation.

### 2.4 Functional enrichment analysis

Functional annotation and pathway enrichment analyses were performed on the intersection gene set. Gene symbols were first converted to human Entrez IDs using the org. Hs.e.g.,.db package in R, and Gene Ontology (GO) enrichment analysis was conducted with cluster Profiler, covering Biological Process (BP), Molecular Function (MF), and Cellular Component (CC) categories to explore the biological roles of the genes. KEGG (Kyoto Encyclopedia of Genes and Genomes) pathway enrichment analysis was also performed to identify key pathways closely associated with disease pathogenesis. Enrichment results were visualized as bar plots using the ggplot2 package, highlighting significantly enriched GO terms and KEGG pathways and providing a basis for further elucidation of potential molecular mechanisms.

### 2.5 Machine learning

To identify key genes closely associated with UC diagnosis and construct robust predictive models, four machine learning algorithms were applied using R packages: LASSO (Least Absolute Shrinkage and Selection Operator) via glmnet, Support Vector Machine (SVM) via e1071, Random Forest (RF) via random Forest, and K-Nearest Neighbor (KNN) via class. Within the training set (GSE92415), model parameters were optimized using 10-fold cross-validation: LASSO employed cross-validation to select the optimal lambda minimizing prediction error; Random Forest used out-of-bag error estimation; SVM and KNN applied grid search with cross-validated accuracy. The final candidate genes (SPINK5 and SRI) were identified as the intersection across all four algorithms. This conservative intersection strategy prioritizes robustness over sensitivity; genes uniquely identified by individual algorithms may possess biological relevance but were not pursued in this study. Model performance was estimated using repeated 10-fold cross-validation (10 repeats) to reduce optimism bias. The pre-specified model was then applied to the independent validation set (GSE87473) without re-estimation. To enhance robustness and biological consistency, the intersection of genes identified by all four methods was defined as the final candidate gene set.

Model performance was evaluated using receiver operating characteristic (ROC) curves, and the area under the curve (AUC) was calculated to assess diagnostic efficacy. Subsequently, a diagnostic nomogram was constructed using the RMS and Regplot packages. Model reliability and clinical utility were further validated through calibration curves and decision curve analysis (DCA), respectively.

### 2.6 Immune infiltration analysis

To estimate immune cell infiltration in UC tissues, the CIBERSORT algorithm was applied to bulk transcriptomic data. Using the LM22 signature matrix, the relative proportions of 22 immune cell subsets were estimated for each sample, and statistical significance was assessed via permutation testing (perm = 1,000). Results were visualized as stacked bar plots to depict the overall distribution of immune cells across samples, and boxplots were used to compare differences in immune cell infiltration between UC and control groups.

Spearman correlation analysis was performed to evaluate the relationship between the expression levels of key genes and the proportions of infiltrating immune cells. Correlation plots were generated to explore the potential associations between key genes and immune microenvironmental features. Additionally, the linkET package was used to visualize a Spearman correlation matrix heatmap of immune cells, overlaid with connection lines between key genes and immune cell subsets, providing an integrated view of correlation direction and strength. These proportions represent computational estimates rather than direct measurements, and their accuracy depends on the representativeness of the LM22 reference matrix for intestinal mucosa.

### 2.7 Western blot (WB) analysis

Colon tissues were homogenized in RIPA lysis buffer containing a complete protease inhibitor cocktail (Millipore). Protein concentrations were determined using the BCA assay. Equal amounts of protein were separated on 10% SDS-PAGE gels and transferred onto PVDF membranes (Bio-Rad). Membranes were incubated with primary antibodies overnight at 4 °C, followed by incubation with appropriate secondary antibodies at room temperature for 2 h. Signal detection was performed using an enhanced chemiluminescence (ECL) kit. All Western blot experiments were performed using independent biological samples (n = 3 per group), and each experiment was repeated at least three times to ensure reproducibility. Band intensities were quantified by densitometric analysis and normalized to GAPDH as an internal control.

### 2.8 Immunohistochemistry (IHC)

For IHC, 4 µm paraffin-embedded tissue sections were subjected to endogenous peroxidase blocking, antigen retrieval, and blocking steps. Sections were then incubated with primary antibodies at 4 °C overnight, followed by incubation with biotinylated secondary antibodies at room temperature for 1–2 h. Immunostaining was visualized using a DAB chromogenic kit. Two independent pathologists blinded to sample identity performed scoring. Inter-observer agreement: Cohen’s kappa = 0.85.

### 2.9 Quantitative real-time PCR (qPCR)

Total RNA was extracted from colon tissues using TRIzol reagent, and cDNA was synthesized according to the manufacturer’s instructions. Quantitative real-time PCR was performed on a CFX96 system using SYBR Green Premix Ex Taq. Primer sequences have been added: SPINK5 Forward: 5′-AGC​TGC​TGA​TCC​TGG​TGC​TA-3′, SPINK5 Reverse: 5′-GGT​GGT​GGT​GAT​GAT​GGT​GG-3′, SRI Forward: 5′-CAG​CAG​CAG​CAG​CAG​CAG​CA-3′, SRI Reverse: 5′-AGC​AGC​AGC​AGC​AGC​AGC​AG-3′, GAPDH Forward: 5′-GGA​GCG​AGA​TCC​CTC​CAA​AAT-3′, GAPDH Reverse: 5′-GGC​TGT​TGT​CAT​ACT​TCT​CAT​GG-3'. Cycling conditions: 95 °C for 30s, followed by 40 cycles of 95 °C for 5s and 60 °C for 30s, relative quantification using the 2^(-ΔΔCt)^ method.

### 2.10 Statistical analysis

Data are presented as mean ± standard deviation (mean ± SD). One-way analysis of variance (one-way ANOVA) was used to compare differences between groups. Statistical analyses were performed using GraphPad Prism 8 (GraphPad Software), and p < 0.05 was considered statistically significant.

## 3 Results

### 3.1 Single-cell sequencing analysis

**FIGURE 1:**
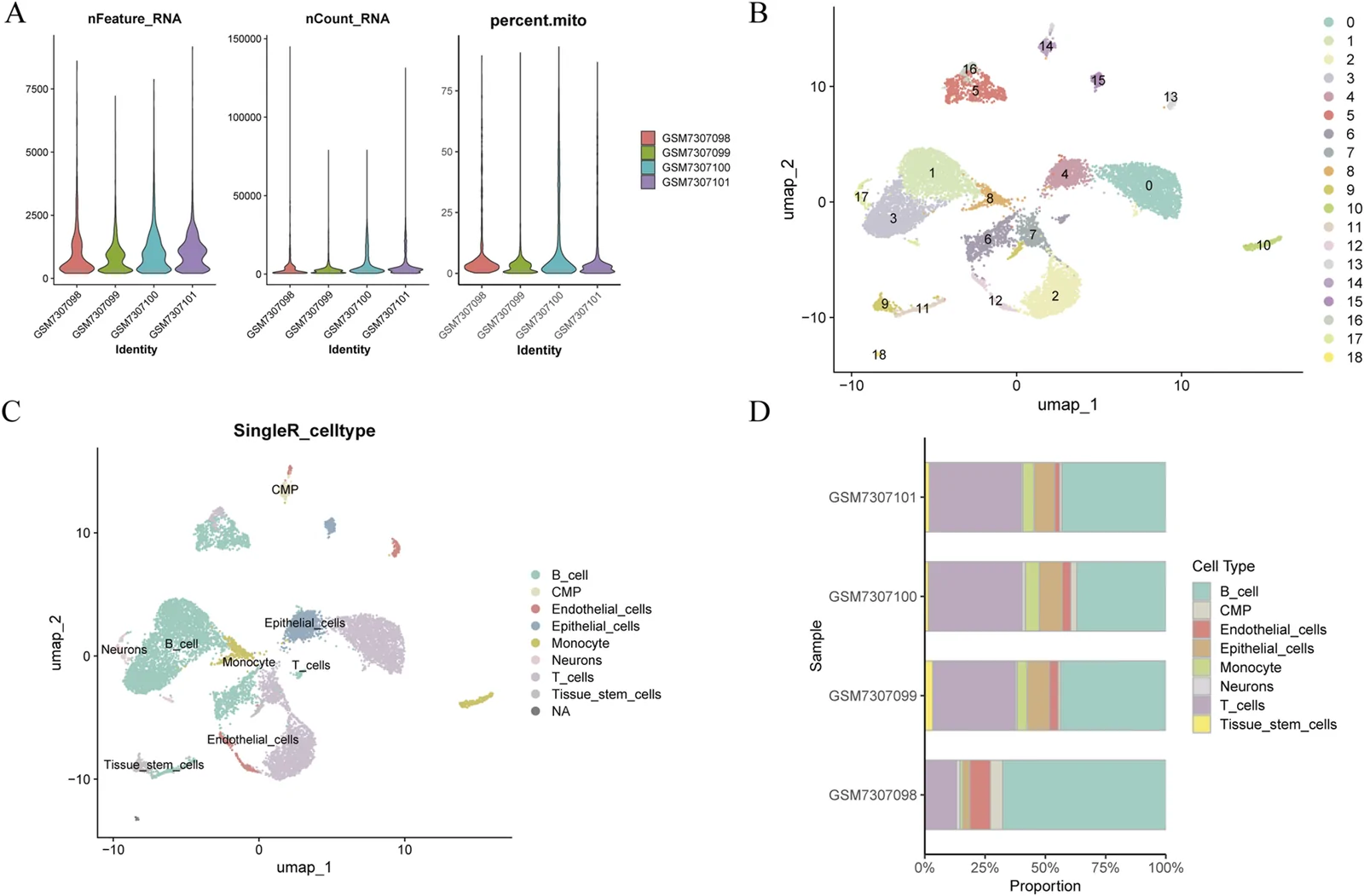
Subpopulation identification and cell annotation in UC samples. **(A)** Violin plots showing the distributions of nFeature_RNA, nCount_RNA, and percent. Mito across different samples. **(B)** UMAP plot illustrating cell distribution in low-dimensional space. **(C)** UMAP visualization of cell type classification results based on the SingleR method. **(D)** Bar plots showing the proportions of immune cell types across different samples.

We first performed stringent quality control and filtering on the single-cell RNA-seq data from UC samples. Genes expressed in fewer than five cells were excluded to remove low-expression or noisy genes, and only cells detecting at least 200 genes were retained to ensure sufficient sequencing depth. Further filtering retained high-quality cells with more than 300 detected genes while excluding cells with mitochondrial gene content exceeding 20%, effectively removing low-quality or potentially apoptotic cells. The distributions of nFeature_RNA, nCount_RNA, and percent. mito across samples are shown in Figure 1A.

Clustering analysis was performed with a resolution of 0.6, identifying 18 subclusters (Figure 1B). Cell type annotation using SingleR classified these subclusters into eight major cell types (Figure 1C): B cells (B_cell), common myeloid progenitors (CMP), endothelial cells (Endothelial_cells), epithelial cells (Epithelial_cells), monocytes (Monocyte), neurons (Neurons), T cells (T_cells), and tissue stem cells (Tissue_stem_cells). The proportions of each cell type across different UC samples were calculated and visualized (Figure 1D), and gene expression profiles within each cell type were analyzed. Given the pivotal role of epithelial cells in UC pathogenesis, they were selected as the primary focus of this study, and 2,215 epithelial cell–related genes were identified.

### 3.2 Identification and functional characterization of epithelial cell–related UC genes

**FIGURE 2:**
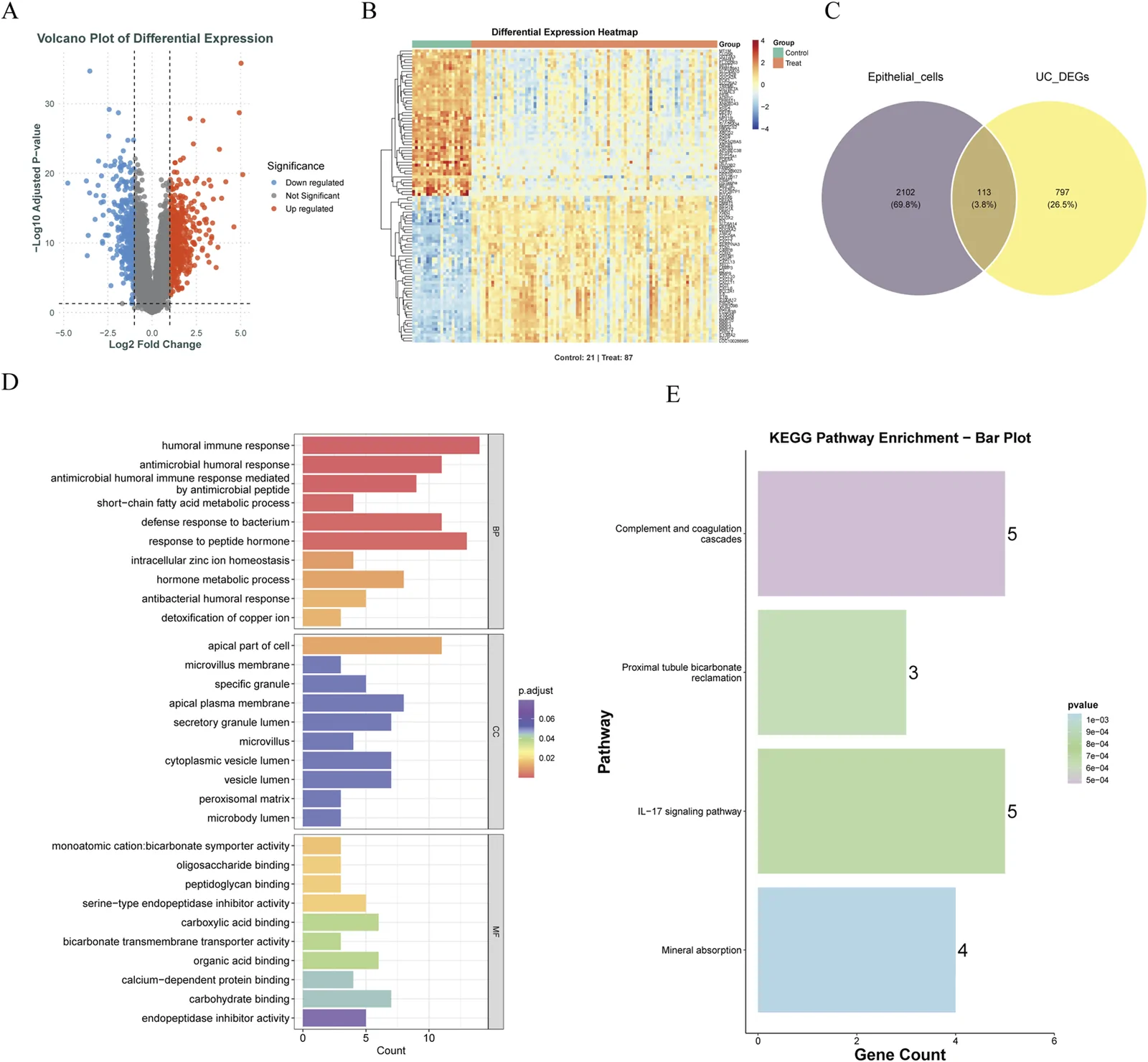
Differential expression analysis and pathway enrichment. **(A)** Volcano plot displaying the distribution of differentially expressed genes (DEGs). **(B)** Heatmap of DEGs. **(C)** Venn diagram showing the overlap between epithelial cell–related genes and UC DEGs. **(D)** Bar plot of Gene Ontology (GO) enrichment analysis for intersection genes. **(E)** Bar plot of KEGG pathway enrichment analysis for intersection genes.

To explore the relationship between epithelial cell–related genes and UC, differential expression analysis was performed on the integrated gene matrix. A total of 910 genes were identified as differentially expressed between UC and normal samples, including 575 upregulated and 335 downregulated genes. These DEGs were visualized using a volcano plot (Figure 2A) and heatmap (Figure 2B).

Intersection analysis using a Venn diagram (Figure 2C) between the 910 DEGs and 2,215 epithelial cell–related genes yielded 113 epithelial cell–associated DEGs.

To further elucidate the biological functions of these DEGs, Gene Ontology (GO) and Kyoto Encyclopedia of Genes and Genomes (KEGG) enrichment analyses were performed. GO enrichment revealed that, in the BP category, these genes were mainly involved in the humoral immune response, antimicrobial humoral response, and antimicrobial humoral immune response mediated by antimicrobial peptides; in the CC category, they were primarily associated with the apical part of the cell; and in the MF category, they were enriched for monoatomic cation:bicarbonate symporter activity, oligosaccharide binding, and peptidoglycan binding (Figure 2D). KEGG pathway analysis indicated that these genes were significantly enriched in complement and coagulation cascades, proximal tubule bicarbonate reclamation, IL-17 signaling pathway, and mineral absorption (Figure 2E).

### 3.3 Construction of an epithelial cell–related UC diagnostic model using machine learning

**FIGURE 3:**
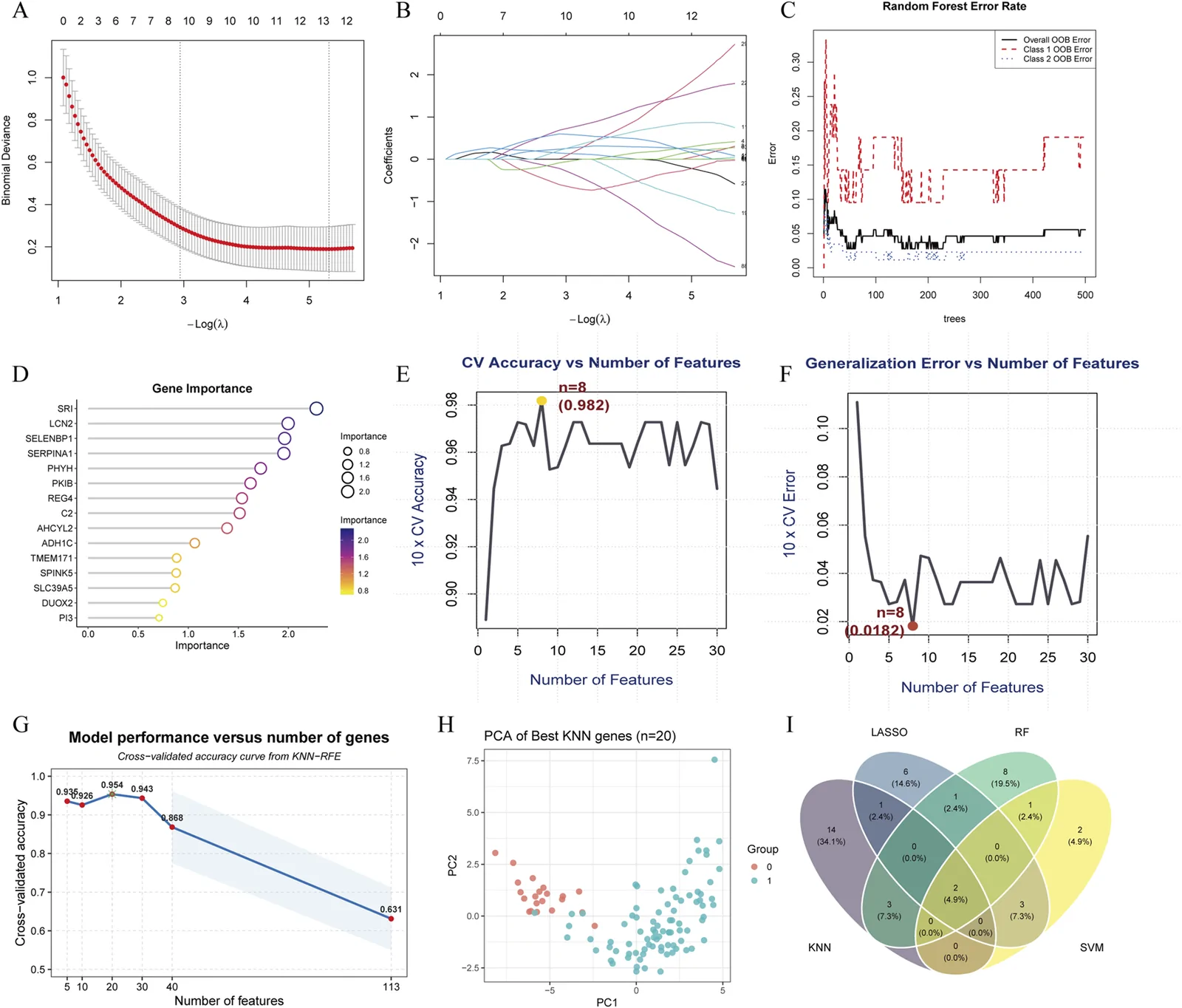
Performance evaluation and feature selection of machine learning models. **(A–H)** Construction of UC diagnostic models using LASSO, RF, SVM, and KNN algorithms, highlighting key features and model performance. **(I)** Venn diagram showing overlapping genes identified across different models (LASSO, RF, KNN, SVM).

We constructed a UC diagnostic model based on epithelial cell–related genes using multiple machine learning algorithms. LASSO analysis (Figures 3A,B) identified 13 key genes closely associated with disease diagnosis: PLA2G2A, OLFM4, SRI, PDZK1IP1, TST, ASRGL1, SPINK5, S100P, DMBT1, HOXA9, PHYH, GPX8, and SEC24D.

Random Forest (RF) analysis showed that both overall and class-specific error rates improved as model training progressed, eventually reaching optimal values (Figure 3C). Among the 113 intersection genes, the 15 genes with the highest feature importance scores were identified: SRI, LCN2, SELENBP1, SERPINA1, PHYH, PKIB, REG4, C2, AHCYL2, ADH1C, TMEM171, SPINK5, SLC39A5, DUOX2, and PI3 (Figure 3D).

Support Vector Machine (SVM) analysis indicated that optimal diagnostic performance was achieved with a model including eight genes, yielding minimal error and an accuracy of 0.982 (Figures 3E,F). These genes were ASRGL1, HMGCS2, ZG16B, SRI, PDZK1IP1, HOXA9, SPINK5, and SELENBP1. K-Nearest Neighbor (KNN) analysis demonstrated that cross-validation accuracy varied with the number of genes. Maximum accuracy (0.954) was achieved with 20 features, while additional features led to decreased performance (Figure 3G). Principal component analysis (PCA) of the 20 selected genes showed clear separation between groups in the principal component space, indicating that these genes effectively distinguish samples (Figure 3H).

**FIGURE 4:**
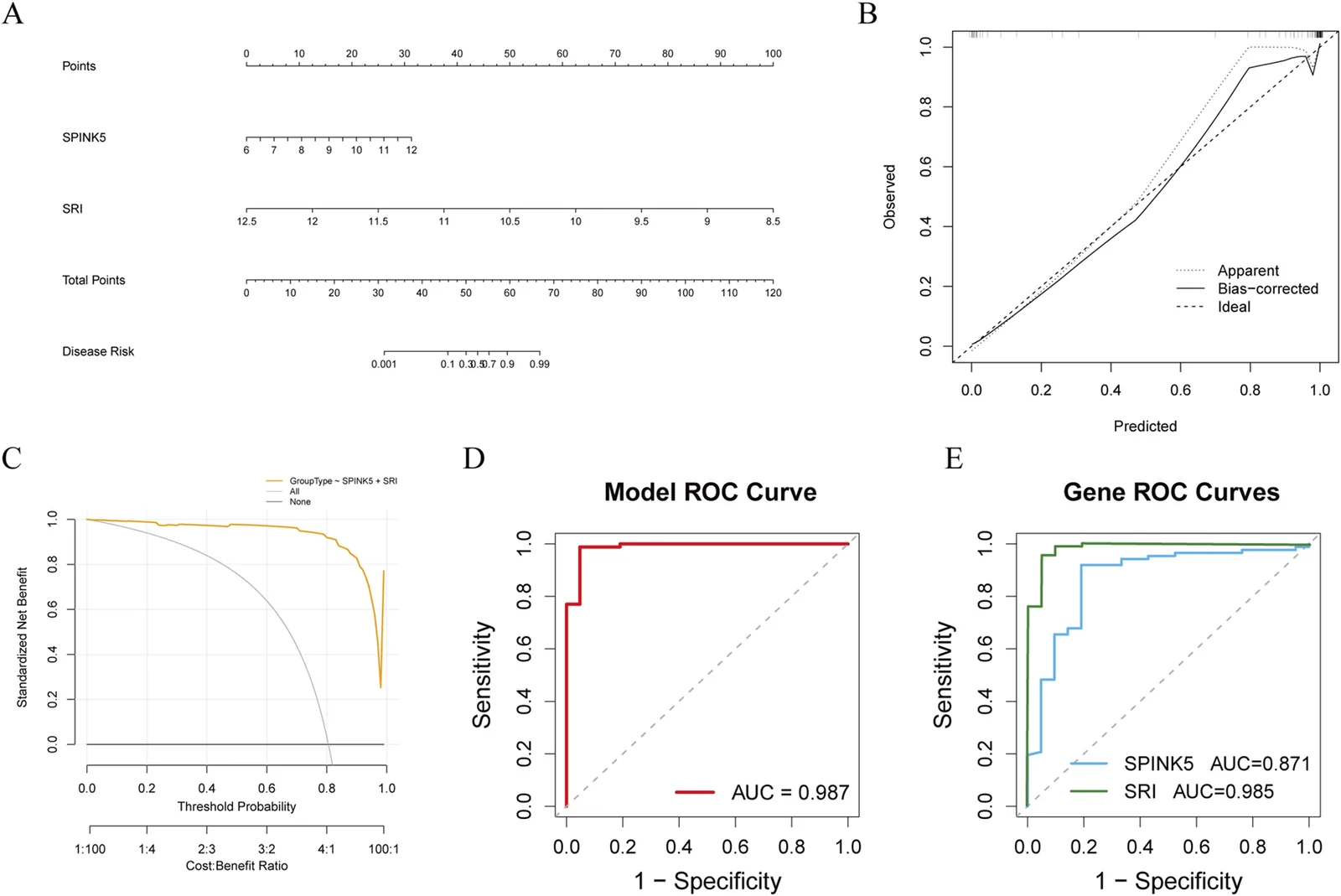
Validation and performance assessment of the diagnostic model in the training set. **(A)** Nomogram for predicting UC probability based on key gene expression. **(B, C)** Calibration and decision curve analyses evaluating model accuracy and clinical utility. **(D)** ROC curve of the diagnostic model. **(E)** ROC curves of the key genes SPINK5 and SRI.

To identify shared key genes across algorithms, a Venn diagram analysis revealed two intersection genes: SPINK5 and SRI (Figure 3I). A nomogram was constructed to further evaluate the diagnostic capability of the model for UC (Figure 4A). Calibration curves indicated minimal differences between observed and predicted UC risk, demonstrating high model accuracy (Figure 4B). Decision curve analysis (DCA) showed that patients could derive substantial net benefit, suggesting potential clinical applicability of the model (Figure 4C). ROC analysis further confirmed excellent diagnostic performance, with an AUC of 0.987. Individually, SPINK5 and SRI achieved AUCs of 0.871 and 0.985, respectively, demonstrating strong diagnostic efficacy.

### 3.4 External validation of UC diagnostic model

**FIGURE 5:**
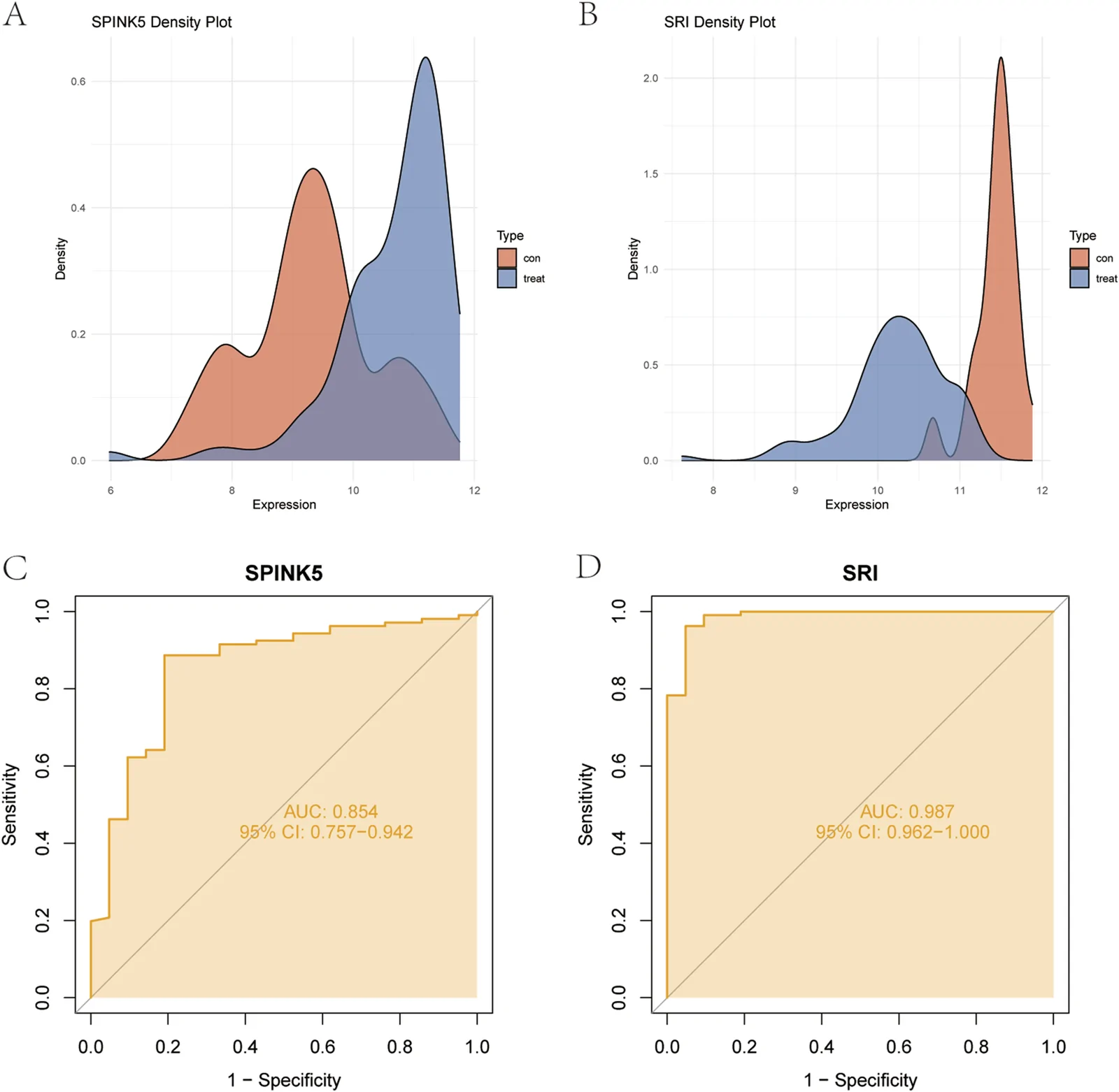
Expression validation and diagnostic performance of key genes in the external validation cohort. **(A)** Expression density plot of SPINK5 in control and UC groups. **(B)** Expression density plot of SRI in control and UC groups. **(C)** ROC curve of SPINK5. **(D)** ROC curve of SRI.

To further validate the diagnostic performance of the key genes, an independent external dataset was used. Differential expression analysis, visualized as a ridge plot, confirmed that SPINK5 was highly expressed in UC, while SRI was downregulated, consistent with trends observed in the training set (Figures 5A,B). ROC curve analysis showed that SPINK5 and SRI achieved AUCs of 0.854 and 0.987, respectively, indicating high accuracy and specificity for UC diagnosis in the external cohort (Figures 5C,D). The exceptionally high AUC for SRI (0.987) reflects its marked and consistent downregulation in UC with low inter-sample variance, creating a near-bimodal distribution. However, this performance should be interpreted cautiously given the potential for cohort-specific effects and platform compatibility between training and validation sets.

### 3.5 Immune infiltration landscape in UC patients

**FIGURE 6:**
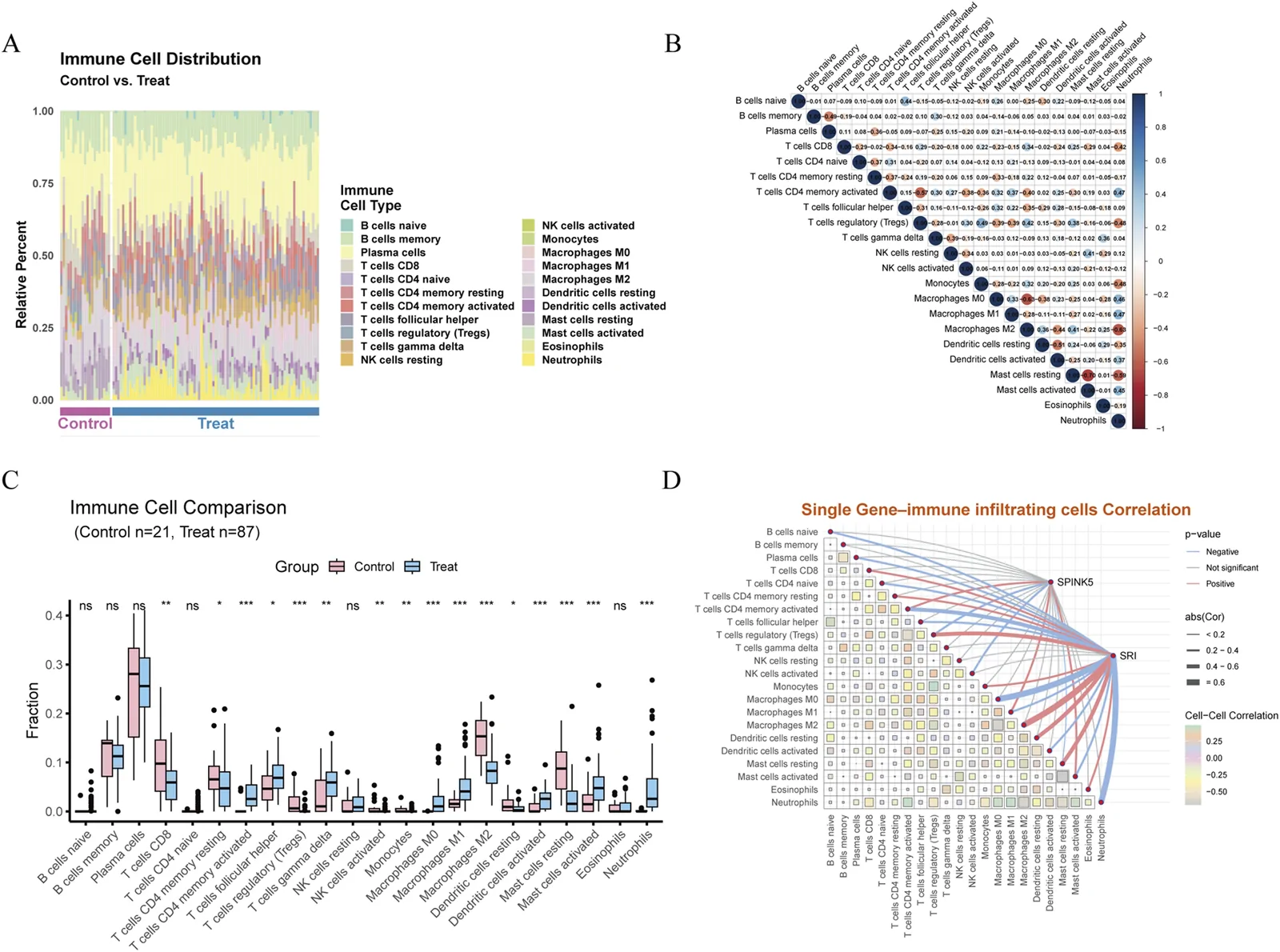
Immune cell distribution and correlation analysis. **(A)** Relative proportions of immune cell types in control and UC groups. **(B)** Correlation heatmap among immune cell types. **(C)** Boxplots showing differences in proportions of immune cell types between control and UC groups, with significance indicated by asterisks. **(D)** Correlation analysis between individual key genes and immune cell infiltration.

We applied CIBERSORT to explore differences in 22 immune cell types in UC patients. Stacked bar plots (Figure 6A) illustrated the composition of immune cells in control and UC groups. A correlation heatmap (Figure 6B), based on Spearman correlation coefficients, visualized relationships among different immune cell types.

Boxplot analysis (Figure 6C) revealed that the proportions of CD4 memory activated T cells, follicular helper T cells, gamma delta T cells, M0 macrophages, M1 macrophages, activated dendritic cells, activated mast cells, and neutrophils were elevated in UC samples, whereas CD8 T cells, CD4 memory resting T cells, regulatory T cells (Tregs), activated NK cells, monocytes, M2 macrophages, resting dendritic cells, and resting mast cells were decreased compared to controls.

Correlation analysis between key genes and immune cells demonstrated that SPINK5 was positively correlated with several immune cell types, including CD4 memory activated T cells, M1 macrophages, and activated mast cells, but negatively correlated with Tregs, activated NK cells, and resting mast cells. Conversely, SRI was positively correlated with CD8 T cells, CD4 memory resting T cells, Tregs, monocytes, M2 macrophages, resting dendritic cells, resting mast cells, and eosinophils, while negatively correlated with naïve B cells, plasma cells, naïve CD4 T cells, CD4 memory activated T cells, follicular helper T cells, M0 macrophages, M1 macrophages, activated dendritic cells, activated mast cells, and neutrophils.

### 3.6 Expression levels of SPINK5 and SRI in colonic mucosa of UC patients

**FIGURE 7:**
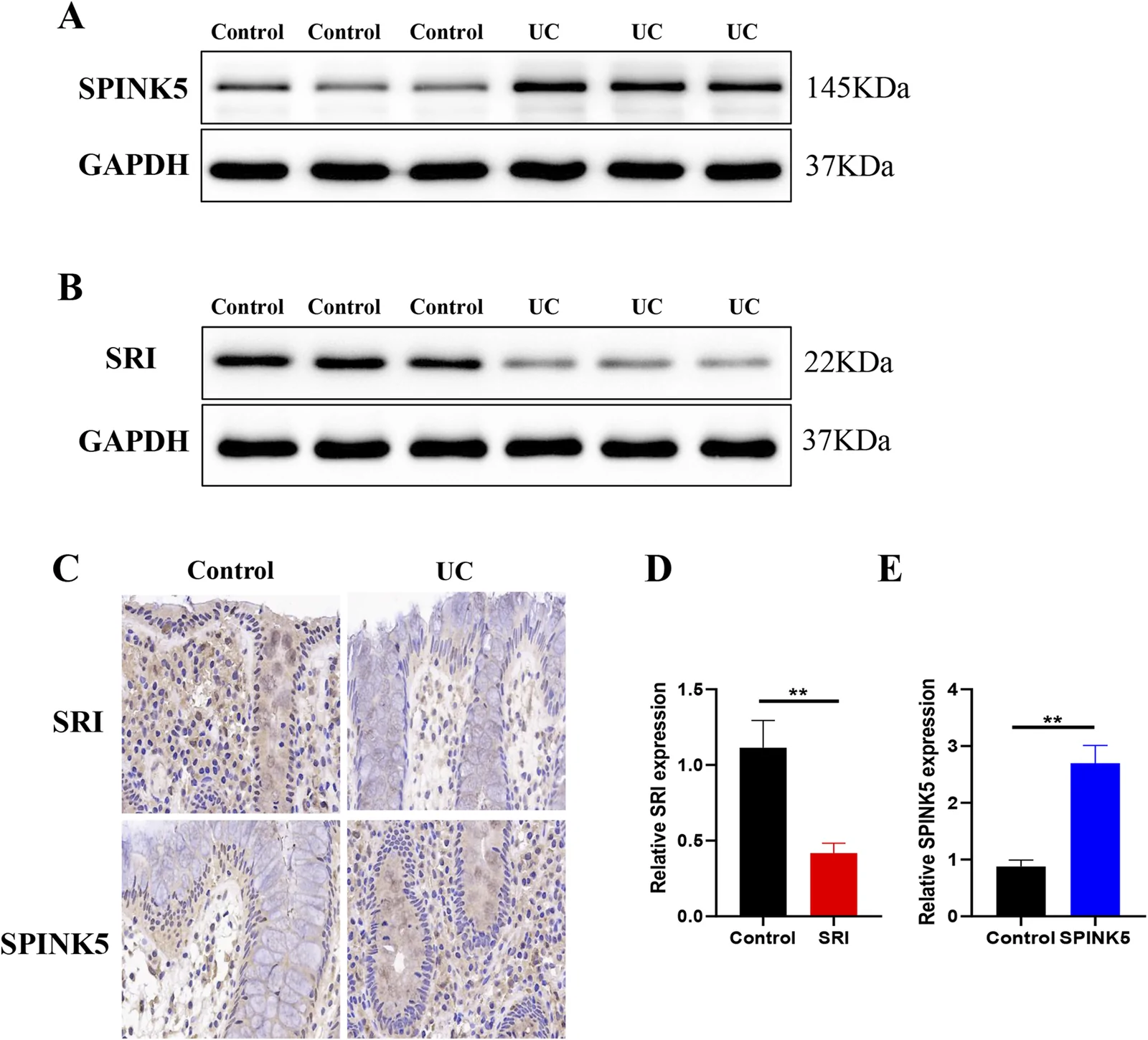
Expression levels of SPINK5 and SRI in colonic mucosa of UC patients. **(A)** Western blot analysis of SPINK5 expression in UC tissues. **(B)** Western blot analysis of SRI expression in UC tissues. **(C)** Immunohistochemistry analysis of SPINK5 and SRI expression in colonic mucosa. **(D)** Quantitative PCR analysis of SRI mRNA expression levels (n = 6). **(E)** Quantitative PCR analysis of SPINK5 mRNA expression levels (n = 6). Data are presented as mean ± SD. *p < 0.05, **p < 0.01, ns, not significant.

We evaluated the expression of SPINK5 and SRI in colonic mucosal tissues from UC patients. Western blot analysis revealed that SPINK5 was upregulated, whereas SRI was downregulated in UC tissues compared to healthy controls (Figures 7A,B). These results were derived from independent biological samples and represent the average of three repeated experiments, supporting the reliability of the observed protein expression differences. Immunohistochemistry further confirmed these findings, showing elevated SPINK5 expression and reduced SRI expression in UC mucosa (Figure 7C). Quantitative real-time PCR analysis validated these expression patterns at the mRNA level (Figures 7D,E), consistent with the protein-level results. Given the small sample size (n = 6 per group), we report observed effect sizes (SPINK5: Cohen’s d = 1.85; SRI: Cohen’s d = 1.62) and their 95% confidence intervals rather than relying solely on statistical significance. These large effect sizes support the biological relevance of the findings, but confirmation in larger cohorts is essential.

## 4 Discussion

This study systematically integrated single-cell transcriptomics, bulk RNA-seq, machine learning algorithms, and histological and molecular validations to identify, from an epithelial cell-associated perspective, the key diagnostic molecules SPINK5 and SRI in ulcerative colitis (UC), and to construct an exploratory gene-based diagnostic model. This approach not only highlights the central role of epithelial cells in UC pathogenesis but also underscores the advantages of integrating multi-dimensional data and multiple algorithms for biomarker discovery.

Our single-cell RNA-seq analysis provided cell-type-level information on epithelial cells in UC patients”, consistent with recent single-cell studies demonstrating epithelial cell heterogeneity and functional shifts in inflammatory bowel disease (IBD). Conventional bulk RNA analyses are limited in distinguishing different cell types, whereas intersecting epithelial-specific genes with differentially expressed genes allowed us to identify a candidate genes that may improve the cell-type relevance of candidate gene selection.

In machine learning-based screening, SPINK5 and SRI were consistently identified across multiple algorithms (e.g., LASSO, Random Forest), which aligns with other studies employing machine learning to analyze UC transcriptomes for diagnostic modeling. SPINK5, as a serine protease inhibitor, may contribute to maintaining epithelial barrier integrity by regulating protease-mediated epithelial damage. Its upregulation in UC may represent a compensatory protective response to ongoing inflammation and barrier disruption. In addition, its positive correlation with pro-inflammatory immune cells suggests that SPINK5 may participate in shaping the inflammatory microenvironment and amplifying immune responses (Wang J. et al., 2024; Genovese et al., 2020; Xu et al., 2025). In contrast, SRI is involved in calcium homeostasis and cellular stress regulation. Its downregulation in UC may impair epithelial cell resistance to inflammatory and oxidative stress, thereby exacerbating epithelial dysfunction. Given the essential role of calcium signaling in epithelial repair and survival, decreased SRI expression may contribute to impaired mucosal healing and persistent inflammation in UC (Tito et al., 2025; Gong et al., 2024; Erkert et al., 2026).

CIBERSORT-based immune infiltration analysis estimated that pro-inflammatory cell types, including activated CD4 memory T cells, M1 macrophages, and neutrophils, were increased in UC mucosa, while regulatory cell types such as Tregs and M2 macrophages were reduced (von Locquenghien et al., 2025; Castro-Dopico et al., 2019; Wang Y. et al., 2024). This is consistent with previous single-cell and spatial transcriptomics studies describing microenvironment remodeling in IBD, including spatial heterogeneity of macrophage and neutrophil populations. Differential correlations of SPINK5 and SRI with these immune cell subsets further support their role as key nodes within the epithelial–immune interaction network.

The term ‘crosstalk’ implies bidirectional signaling between epithelial and immune cells. While our CIBERSORT analysis reveals statistically significant correlations between SPINK5/SRI expression and immune cell proportions, these data do not demonstrate direct cell-cell communication. Experimental approaches such as epithelial-immune co-culture assays, conditioned medium transfer experiments, or spatial transcriptomics are needed to validate functional crosstalk.

The strategy used in this study provides a conservative screening framework by combining epithelial cell-associated gene selection, multiple machine learning algorithms, and external validation. This design is consistent with recent trends integrating single-cell analysis and machine learning for disease mechanism elucidation and diagnostic model construction. This approach minimizes algorithmic and platform bias, improving the biological interpretability and translational potential of candidate genes (Townsend et al., 2024).

Despite these significant findings, certain limitations remain. First, our analyses and model training primarily relied on publicly available datasets, which may be affected by batch effects, sample heterogeneity, and incomplete clinical information. Prospective, multi-center clinical cohorts are needed to validate model stability and determine clinical thresholds. Second, although SPINK5 and SRI expression was confirmed at the tissue level via Western blot, immunohistochemistry, and qPCR, their causal roles in epithelial barrier function, inflammation, and cell survival have not yet been systematically investigated *in vivo* or *in vitro*. Moreover, the current model focuses on differentiating UC from healthy controls, without assessing its utility in distinguishing UC from Crohn’s disease, evaluating disease activity, or predicting treatment response or relapse risk.

Future studies could explore the functional roles of SPINK5 and SRI in epithelial–immune co-culture systems or mouse IBD models to elucidate their involvement in epithelial repair, inflammation regulation, and barrier restoration. Additionally, integrating single-cell and spatial transcriptomics may reveal the spatial expression patterns of these genes within tissue microenvironments and their localization relative to immune cells, a strategy that has shown promise in IBD research. Finally, constructing more comprehensive subtype or predictive models based on machine learning—incorporating metabolic pathways, apoptosis/cuproptosis, or intracellular signaling pathways, analogous to cuproptosis-related gene subtyping—may further enhance UC molecular classification and precision medicine capabilities (Tang et al., 2023; Huang et al., 2025; Liu et al., 2025; Yao et al., 2026; Gudiño et al., 2025).

In conclusion, this study identified epithelial-specific DEGs through single-cell analysis, applied machine learning to screen and validate SPINK5 and SRI, and integrated immune infiltration analysis and clinical tissue validation to propose SPINK5 and SRI as potential epithelial cell-associated diagnostic candidates related to epithelial dysfunction. These findings provide new insights into UC pathogenesis and offer potential targets for diagnostic and therapeutic strategies based on epithelial–immune interactions. It is important to emphasize that the associations between SPINK5/SRI and immune cell subsets identified in this study are correlational. Causal relationships and direct mechanistic interactions require functional validation through *in vitro* co-culture systems, gene manipulation studies, or animal models, which represent important directions for future research.

## 5 Limitations

Several limitations should be acknowledged. First, the single-cell dataset used in this study contained only four UC samples, which may limit the robustness and representativeness of the epithelial cell-associated gene set. Second, external validation was performed in only one independent cohort, and further validation in larger multicenter datasets is required to assess the generalizability of SPINK5, SRI, and the diagnostic model. Third, because detailed clinical variables were not consistently available across the public datasets, the nomogram was constructed only from gene expression data and should be regarded as an exploratory molecular model rather than a comprehensive clinical prediction tool. In addition, CIBERSORT provides inferred immune cell fractions based on the LM22 reference matrix rather than direct measurements of immune cell abundance. Therefore, the immune infiltration results and the high AUC of SRI should be interpreted cautiously and require further confirmation in independent clinical cohorts and experimental studies. The choice of 2,500 highly variable genes and clustering resolution 0.6 was based on standard practice and visual inspection rather than systematic benchmarking. Formal sensitivity analyses comparing alternative parameters were not performed and represent a limitation. Furthermore, the absence of normal control samples in the scRNA-seq dataset precludes direct comparison of epithelial cell states between healthy and diseased mucosa.

## Data Availability

The datasets presented in this study can be found in online repositories. The names of the repository/repositories and accession number(s) can be found below: https://www.ncbi.nlm.nih.gov/geo/ **,** GSE231993 https://www.ncbi.nlm.nih.gov/geo/, GSE92415 https://www.ncbi.nlm.nih.gov/geo/, GSE87473.
