# Correction: Epithelial cell-based multi-omics integration identifies SPINK5 and SRI as novel diagnostic biomarkers for ulcerative colitis

**DOI:** 10.3389/fphar.2026.1928449

**Published:** 2026-09-30

**Authors:** Xingwang Li, Zhanzhen Liu, Zheng Ge, Yuewei Li, Kunhou Yao, Xuhong Lin, Xiaoming Liu, Hongping Xia

**Affiliations:** 1 Department of General Surgery, The First Affiliated Hospital of Xi’an Jiaotong University, Xi’an, China; 2 Department of General Surgery (Colorectal Surgery), Huaihe Hospital Affiliated to Henan University, Kaifeng, China; 3 Department of General Surgery (Colorectal Surgery), The Sixth Affiliated Hospital, Sun Yat-sen University, Guangzhou, China; 4 Guangdong Provincial Key Laboratory of Colorectal and Pelvic Floor Diseases, The Sixth Affiliated Hospital, Sun Yat-sen University, Guangzhou, China; 5 Biomedical Innovation Center, The Sixth Affiliated Hospital, Sun Yat-sen University, Guangzhou, China; 6 Department of Gastroenterology, Huaihe Hospital Affiliated to Henan University, Kaifeng, China; 7 Zhongda Hospital, School of Medicine, Advanced Institute for Life and Health, Southeast University, Jiangsu, China

**Keywords:** diagnostic biomarkers, machine learning, serine protease inhibitor kazal type 5, sorcin, ulcerative colitis

Author [Xiaoming Liu] was erroneously [included] as corresponding author. The correct corresponding author(s) is [Hongping Xia, 101013473@seu.edu.cn].

The original version of this article has been updated.

